# Dl-3-n-Butylphthalide promotes neovascularization and neurological recovery in a rat model of intracerebral hemorrhage

**DOI:** 10.1186/s12868-020-00575-3

**Published:** 2020-05-29

**Authors:** Ewen Tu, Qiong Chen, Li Tan, Yan Wang

**Affiliations:** 1 Department of Neurology, Hunan Brain Hospital, Hunan University of Chinese Medicine, Changsha, 410208 Hunan China; 2 Department of Neurology, The Fourth Hospital of Changsha, Changsha, 410006 Hunan China

**Keywords:** Angiogenesis, Neurorehabilitation, Neurovascular unit, Phytotherapy, Stroke

## Abstract

**Background:**

Cerebral stroke occurs following ischemic and hemorrhagic lesions in the brain. Survival and recovery of stroke patients depend on the severity of the initial injury but also the therapeutic approaches applied for emergent lifesaving and continuing post-stroke management. Dl-3-n-Butylphthalide (NBP), an active compound derived from Chinese celery seeds, has shown clinical efficacy in the treatment of ischemic cerebral stroke.

**Results:**

In the present study we explored the therapeutic effect of NBP in a rat model of intracerebral hemorrhage (ICH), focusing on its potential role in promoting neovascularization in the perihemorrhagic zone. ICH was induced in male Sprague-Dawley rats by unilateral injection of autologous blood into the globus pallidus, with sham-operated (Sham group), vehicle-treated (ICH) and NBP-treated (at 10 and 25 mg/kg/Bid, p.o., ICH + NBP10 and ICH + NBP25, respectively) groups examined behaviorally, macroscopically, histologically and biochemically at 1, 3, 7 and 15 days (d) post operation. Rats in the ICH + NBP10 and ICH + NBP25 groups showed reduced Longa’s motor scores relative to the ICH groups at the 3 and 7d time points, while the hematoma volume was comparable in the two NBP relative to the ICH groups as measured at 7d and 15d. In the perihemorrhagic zone, the numeric density of blood vessels immunolabeled by CD34, an angiogenic marker, was greater in the ICH + NBP10 and ICH + NBP25 than ICH groups, more so in the higher dosage group, at 1, 3, 7 and 15d. Levels of the vascular endothelial growth factor (VEGF) and angiopoietins-2 (Ang-2) proteins were elevated in the NBP groups relative to the sham and vehicle controls in immunoblotting of tissue lysates from the injection region.

**Conclusion:**

These results suggest that NBP can alleviate neurological defects following experimentally induced local brain hemorrhage, which is associated with a potential role of this drug in promoting neovascularization surrounding the bleeding loci.

## Background

Intracerebral hemorrhage (ICH) refers to primary non-traumatic parenchymal bleeding that often onsets suddenly and manifests as acute loss of broad neurological and cognitive functions. All over the world, ICH remains associated with high morbidity and mortality, and represents one of the greatest healthcare crises affecting the quality of people’s life [1–5]. ICH is the common (consisting of 10–15% of all stroke cases) but most severe subtype of cerebral stroke, which also includes the cases suffered from ischemic and other insults [6–9]. Much progress has been made in recent years in understanding the pathogenesis of ICH [7, 10]. However, unfortunately, none of the current clinical interventions, including aggressive blood pressure control, homeostasis management and platelet transfusion, have significantly improved the outcome of cerebral stroke among patients [11–16].

Besides a space-occupying effect, the hematoma formed during ICH appears to rapidly impair regional blood flow, and thus causing an ischemic effect to the brain tissue adjacent to the bleeding site [17]. The damage in the above so-called perihemorrhagic zone (PHZ) can aggravate neuronal damage and neurological deficits. According to the concept of neurovascular unit (NVU) treatment in management of cerebral stroke, measures to promote angiogenesis are recommended to apply as early as possible when a stroke patient receives medical attention [18–22]. It is considered that, by stimulating angiogenesis in the tissue adjacent to the hematoma, microvascular perfusion and oxygen supply would be improved locally, while the blood–brain barrier is stabilized. A better functioning microcirculation may play a crucial neuroprotective role, thereby improve the neurological recovery [23–27].

The practice of traditional Chinese medicine in the care of patients in China has improved the identification and development of many natural or herbal medicines in clinical management of stroke [28–31]. Among many, an active component of Chinese celery seeds, chemically identified as Dl-3-n-Butylphthalide (NBP), has been recently shown as a promising neuroprotective medicine for the treatment of cerebral stroke and other neurological diseases [32–35]. This compound has been approved by the China Food and Drug Administration for prescription to patients with ischemic brain stroke since 2002 [36]. Its neuroprotective effects against ischemic cerebral stroke have also been investigated in many animal studies, which appear to be related to multiple cellular and molecular mechanisms [37–45], including a potential proangiogenic efficacy [40, 46]. However, less is known about the therapeutic potential of this compound in experimental models of hemorrhagic cerebral stroke. In the present study, we set out to explore whether NBP administration could alleviate neurological deficits in a rat model of ICH induced experimentally via intrastriatal blood injection. Given the importance of microvascular functioning in neurological recovery, particular efforts were taken to determine if this compound might promote neovascularization around the hemorrhagic site.

## Results

### NBP treatment alleviated neurological deficits in rats with ICH

**Fig. 1:**
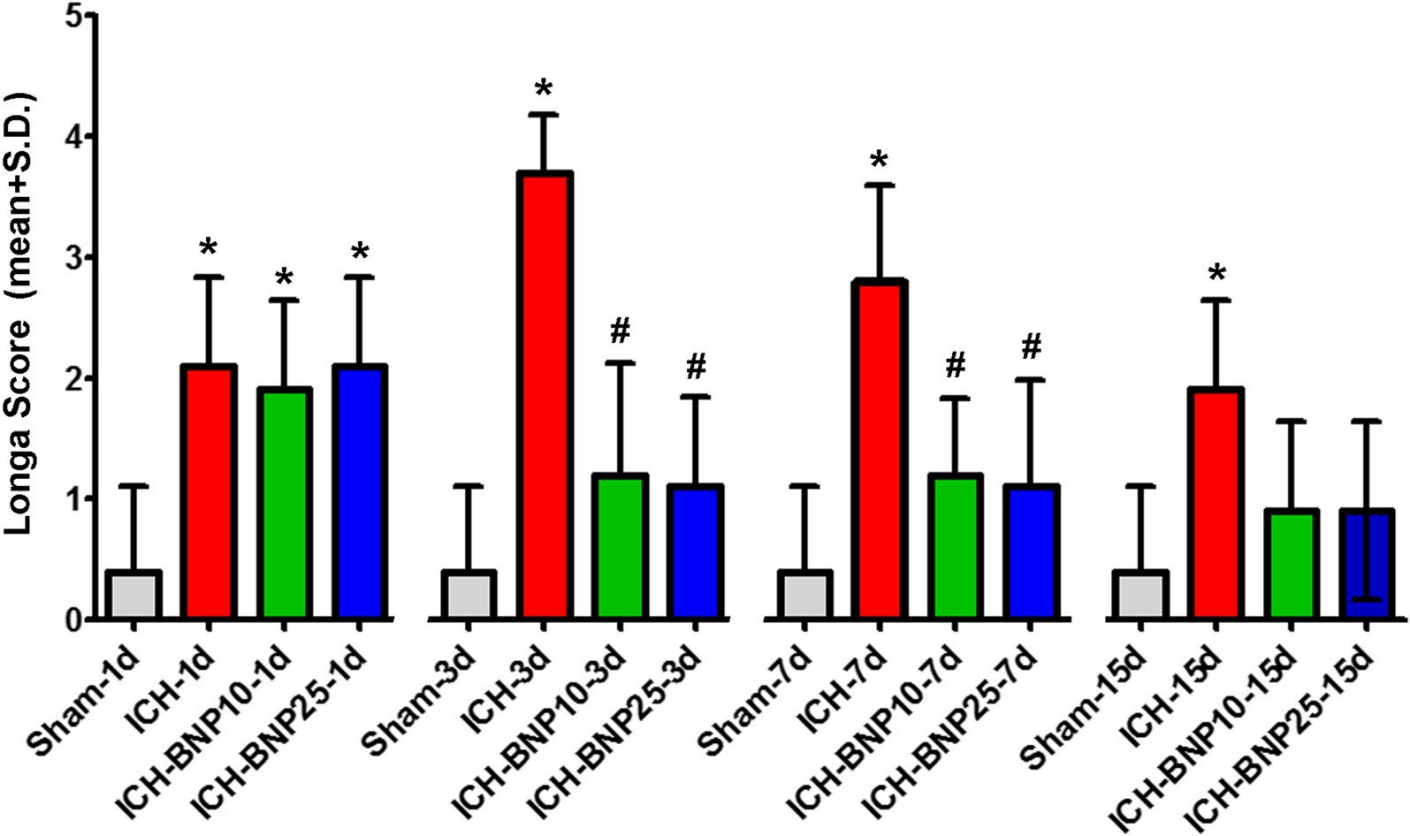
Effect of NBP treatment on neurological performance in a rat model of experimental intracerebral hemorrhage (ICH). Neurological deficit scores are assessed according to the Langa’s scoring method. Scores (mean ± S.D.) recorded at 1, 3, 7 and 15 days (d) post operation are plotted for the sham-operated groups (Sham), ICH model groups treated with vehicle (ICH), and ICH groups treated with NBP at 10 (ICH + NBP10) and 25 (ICH + NBP25) mg/kg Bid. Means are analyzed statistically using ANOVA followed by Bonferroni’s Multiple Comparison Test as post hoc, with *indicating significantly different in comparison to the Sham groups, and # indicating significantly different in comparison to the ICH groups

As reflected by the Longa’s scores, the animal groups subjected to induced ICH showed severe neurological deficits relative to the Sham groups at all surviving time points, with a trend of mitigation in movement inability with the increase of surviving time (Fig. 1). The worst performance in the ICH groups occurred at the 3rd day post operation. The peak of neurological deficiency in the ICH + NBP10 and the ICH + NBP25 groups occurred otherwise at the 1st day post operation. Statistically, the neurobehavioral deficit scores were significantly increased in the ICH groups in comparison with the Sham controls (P < 0.05 between all comparing pairs, ANOVA with Bonferroni’s post hoc test). In both the ICH + NBP10 and ICH + NBP25 groups, the deficits were significantly reduced relative to the ICH (vehicle control) groups at the 3d and 7d time points. However, in the 15d groups, no significant difference remained between the ICH and drug-treated groups, likely owing to the spontaneous recovery of motor functions following the lesion.

### NBP treatment did not reduce hematoma size in rats with ICH

**Fig. 2:**
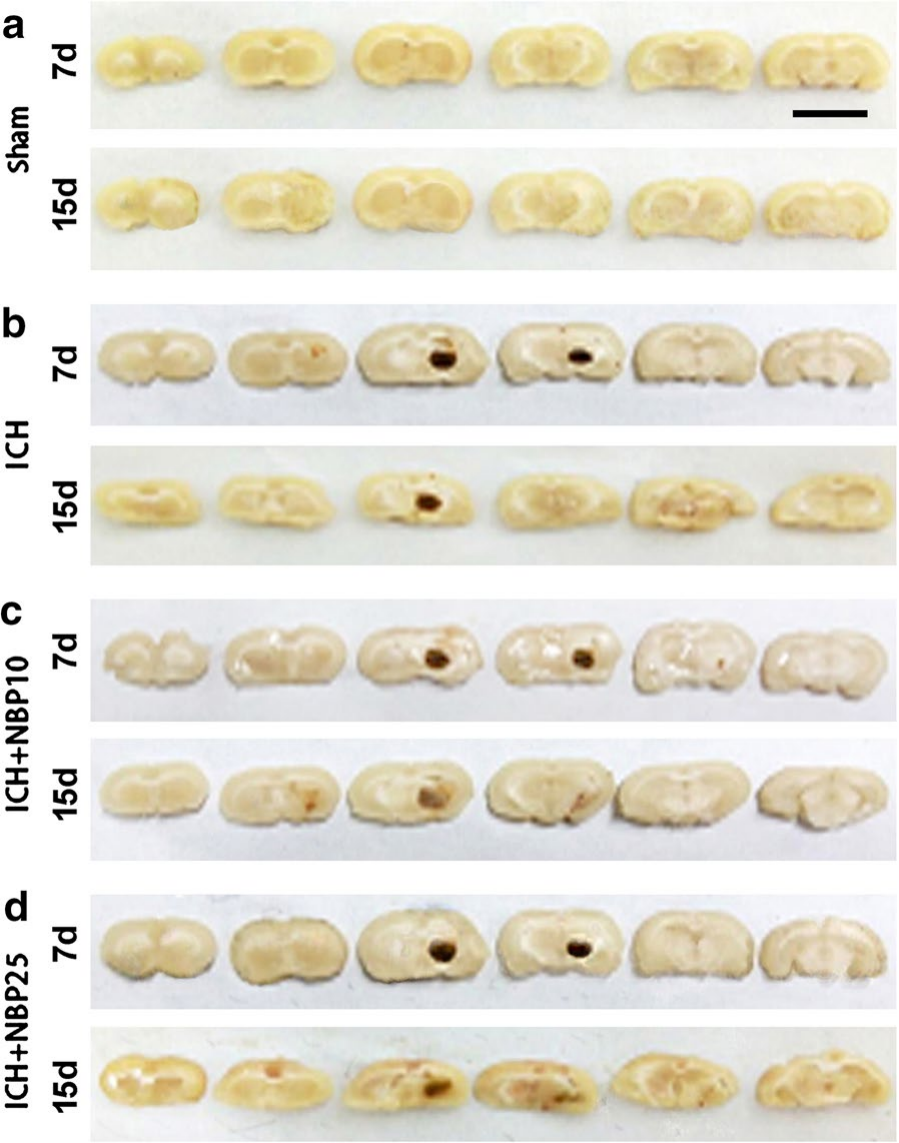
Representative images of frontal consecutive slices of rat brains illustrating the location and area of hematoma in the four groups of animals surviving 7 and 15 days (**d**) post operation. In the Sham operated brains, no bleeding spots are visible across the frontal to occipital dimension of the brain (**a**). In other groups, a hematoma is seen at the right striatal area most evident in the 3rd and 4th of the 6 slices (**b**–**c**). Scale bar = 1 cm in (**a**) applying to other panels

Brains from animals surviving 7 d and 15 d post operation were used to measure the hematoma volumes in consecutive coronal slices. On visual inspection, no apparent blood infiltration was seen in the slices from the Sham-operated brains at both time points (Fig. 2a). In contrast, a clear bleeding area was seen around the striatum in the brains from animals subjected to unilateral blood injection (Fig. 2b–d).

**Fig. 3:**
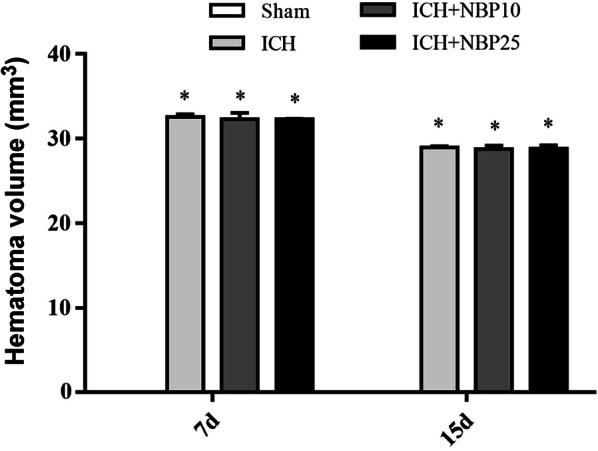
Quantification of the hematoma in a rat model of ICH with and without NBP treatments surviving 7 and 15 day post operation. Means are analyzed statistically using ANOVA followed by Bonferroni’s Multiple Comparison Test as post hoc. The hematoma volume is minimal in the Sham groups, which is significantly different from the ICH, ICH + NBP10 and ICH + NBP25 groups at 7 and 15 days (d) post operation (n = 5/group/time point, *: P < 0.05). However, no difference exists for the means between each pair of the ICH, ICH + NBP10 and ICH + NBP25 groups

Quantitatively, the hematoma volumes were 32.57 ± 0.31 mm^3^ and 28.99 ± 0.12 mm^3^ in the ICH groups at 7 d and 15 d post operation (n = 5/group/time point), respectively. The values were 32.32 ± 0.72 mm^3^ and 28.80 ± 0.38 mm^3^ in ICH + NBP10 groups, and 32.29 ± 0.06 mm^3^ and 28.81 ± 0.41 mm^3^ in the ICH + NBP25 groups, respectively at the above two time points. Two-way ANOVA analysis showed significant effects related to treatment (P < 0.0001, Df = 3, F = 18070) and time point (P < 0.0001, Df = 3, F = 8095) variations. Bonferroni’s post hoc tests indicated the differences arose from other groups in comparison with the Sham groups (all P < 0.001). Thus, no statistically significant differences existed between each pairing set of the means of the ICH, ICH + NBP10 ICH + NBP25 groups at both time points (P > 0.05) (Fig. 3).

### NBP treatment increased microvascular profiles in rats with ICH

**Fig. 4:**
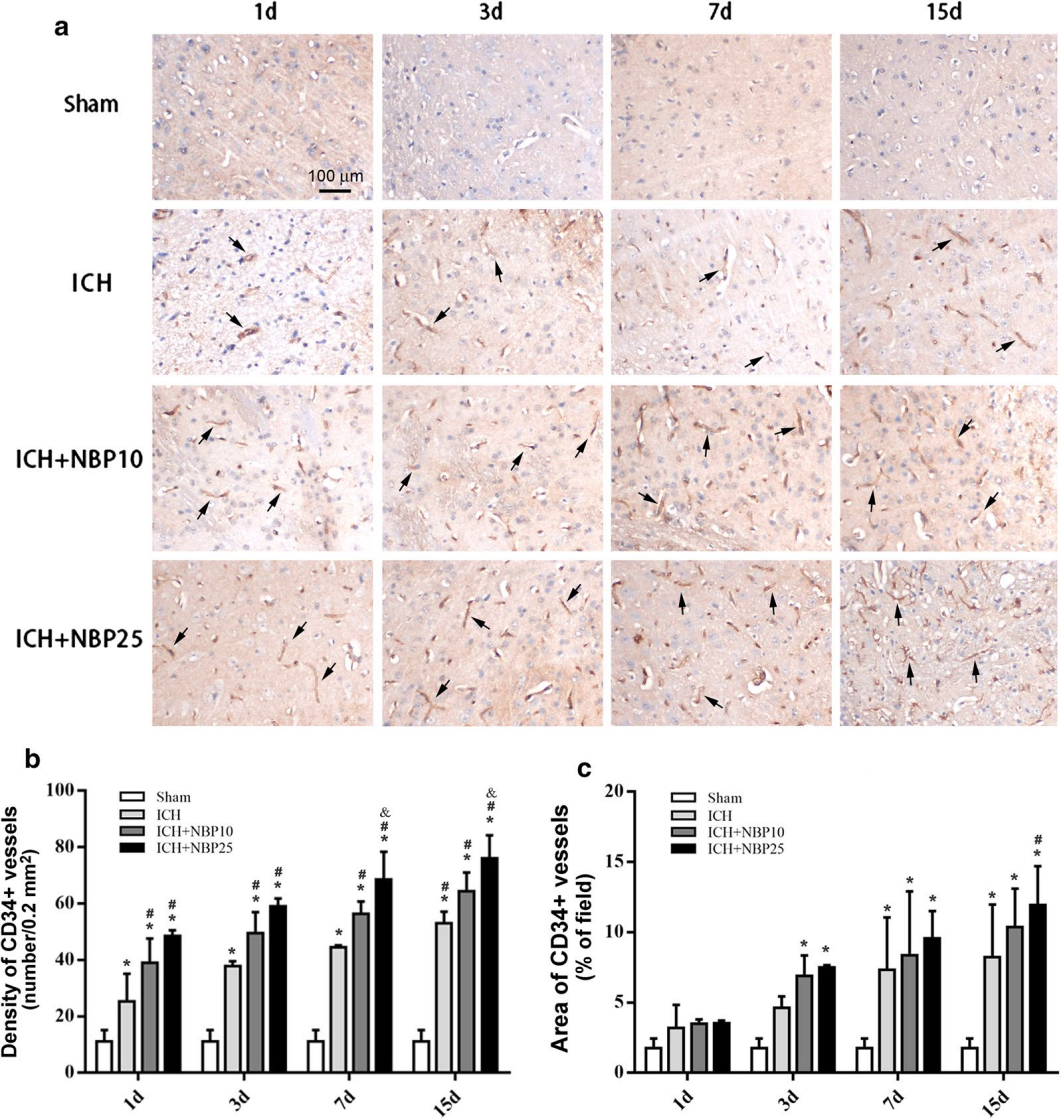
Effect of NBP treatment on putative microvascular formation around the lesion site in a rat model of ICH. Panel (**a**) are micrographs from the striatal injection sites in the brains of representative animals, with the groups and surviving time points as indicated. Shown are immunolabeling with the CD34 antibody with toluidine blue counterstain. Examples of immunolabeled microvascular profiles are indicated by arrows. Panel (**b**) plots the means of the total number of CD34 labeled vascular profiles counted over a 20× microscopical field with an area of 0.2 mm^2^. Panel (**c**) plots the means of fractional area (%) of CD34 labeled vascular profiles. Results from two-way ANOVA followed by Bonferroni’s post hoc tests are marked with symbols. *: Significantly different comparing to the Sham groups; #: Significantly different comparing to the ICH groups; &: Significantly different comparing to the ICH + NBP10 groups. Scale bar = 100 µm in (**a**), applying to all image panels

On microscopical examination, the CD34 antibody labeled very few vascular profiles in the sections prepared from the tissue around the needle track from the Sham operated animals surviving 1d, 3d, 7d and 15d post operation, respectively (Fig. 4a, upper panels). The amount of labeled microvascular structures appeared to be increased in the ICH groups, and further, in the two drug-treated groups surviving to the above time points (Fig. 4a, middle and low panels). Specifically, the sections from the ICH + NBP25 groups at 7d and 15d post operation appeared to contain the highest number of the vascular profiles among all groups (Fig. 4a, low right panels).

The numerical density (mean ± S.D.) of putative new vessels per microscopic field (over a total area of 0.2 mm^2^) was 11.00 ± 4.14 in the Sham groups (combining all time point groups as a purpose for standardization). The values were 25.25 ± 9.91, 37.75 ± 1.78, 44.50 ± 0.71 and 53.00 ± 4.14 in the ICH groups at 1d, 3d, 7d and 15d time points (same order below), respectively; 39.00 ± 8.66, 49.50 ± 7.45; 56.25 ± 4.47 and 64.25 ± 6.78 in the ICH + NBP10 groups; and 48.50 ± 2.02, 59.00 ± 2.83, 68.50 ± 9.90 and 76.00 ± 8.18 groups. Statistically (two-way ANOVA with Bonferroni’s post hoc tests), there existed both time (P < 0.0001, Df = 3, F = 42) and treatment (P < 0.0001, Df = 3, F = 287) dependent significant effects among the means. *Post*-*hoc* tests indicated significant differences for the other groups relative to Sham groups at all time points (P < 0.01 to P < 0.001). There were also differences for the ICH + NBP10 and ICH + NBP25 groups in comparison with ICH groups at all time points (P < 0.05 to P < 0.001). In addition, significant difference was reached between the ICH + NBP10 and ICH + NBP25 groups at 7 (P < 0.01) and 15 d (P < 0.05), respectively (Fig. 4b).

The areal ratios (cross-sectional area of all vascular profiles divided by the field area of the micrograph, expressed as percentage values) were calculated for individual animals and experimental groups. The mean of the vascular fractional area in the ICH groups (combining the 4 time points) was 1.740 ± 0.69%. In the ICH groups, the values were 3.19 ± 1.65%, 4.69 ± 0.80%, 7.33 ± 3.73 and 8.23 ± 3.75% at 1d, 3d, 7d and 15d post operation, respectively. The means in the ICH + NBP10 groups were 3.49 ± 0.32%, 6.90 ± 1.47%; 8.38 ± 4.53% and 10.35 ± 2.76% at the above surviving time points listed in the same order. The values from the ICH + NBP25 groups were 3.52 ± 0.19%, 7.50 ± 0.16%; 9.58 ± 1.92% and 11.93 ± 2.77% at these time points. Thus, the vascular areas showed a trend of increase with the increase of surviving time in all groups subjected to blood injections, with the means higher in the two drug-treated than vehicle groups (P < 0.0001, Df = 3, F = 42 for time effect; P < 0.0001, Df = 3, F = 287 for treatment effect). The means of the ICH groups were significantly higher than that of the Sham groups at 7d and 15d (P < 0.001), and that of the ICH + NBP10 and ICH + NBP25 groups were increased relative to the Sham groups at 3d, 7d and 15d (P < 0.01 to P < 0.001). Among the drug and vehicle-treated groups, the differences in the means did not reach statistical significance (P > 0.05), except for the ICH vs. ICH + NBP25 groups at 15d (P < 0.05) (Fig. 4c).

### NBP treatment elevated VEGF and Ang-2 levels in rats with ICH

**Fig. 5:**
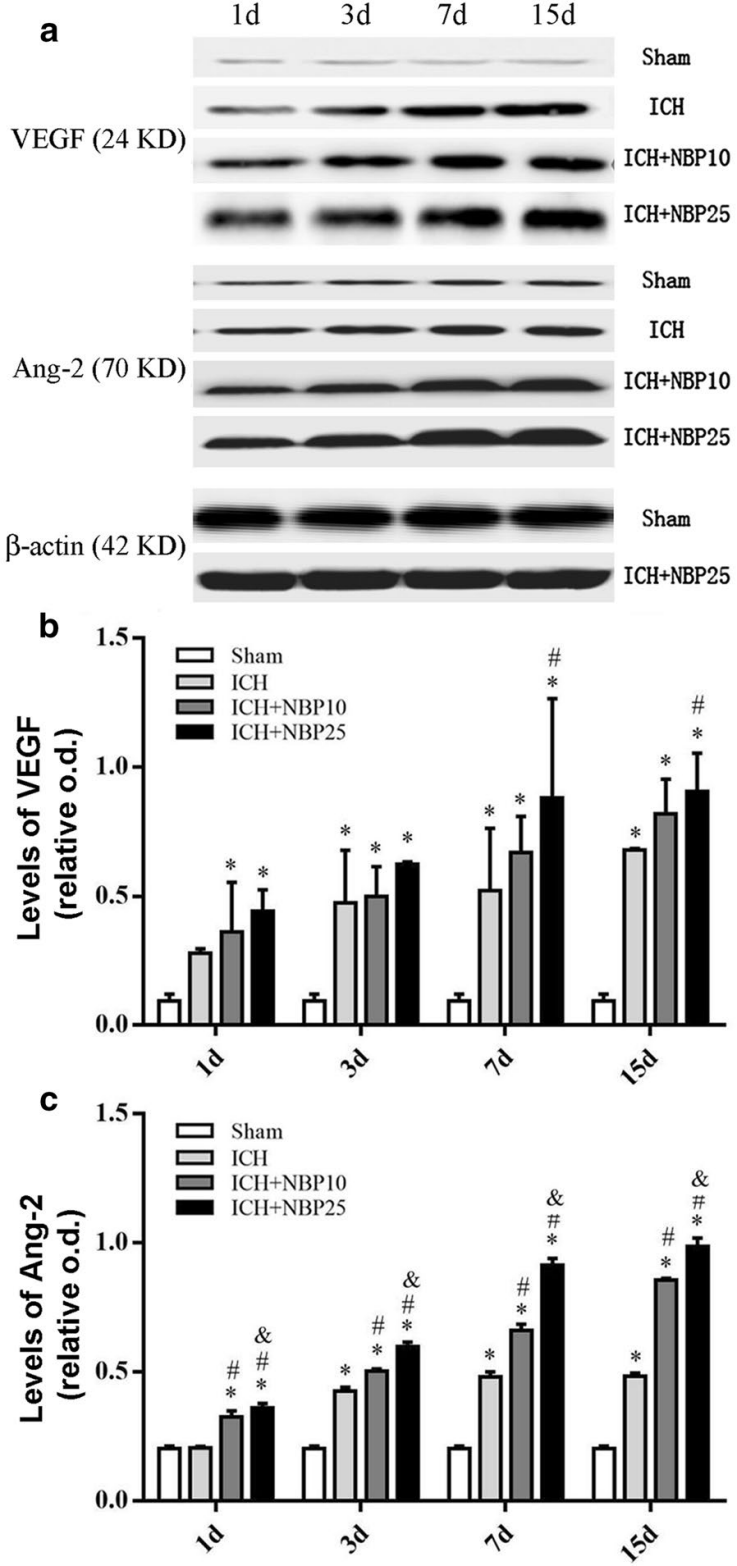
Effect of NBP treatment on the expression of the vascular endothelial growth factor (VEGF) and angiopoietins-2 (Ang-2) proteins in tissue lysates of the injection site in a rat model of ICH. Panel (**a**) shows representative immunoblot images (upper two sets) of VEGF and Ang-2 in the groups as indicated, with representative images (bottom set) showing equal protein loading as blotted with the β-actin antibody. Panels (**b**, **c**) plot the protein levels expressed as relative optic densities (o.d.) normalized to the internal control β-actin. Results from two-way ANOVA followed by Bonferroni’s post hoc tests are marked with symbols, with * indicating significantly different comparing to the Sham groups; # indicating significantly different comparing to the ICH groups; and & indicating significantly different comparing to the ICH + NBP10 groups

To further explore a potential trophic effect of NBP on angiogenesis following ICH, the levels of vascular endothelial growth factor (VEGF) and angiopoietins-2 (Ang-2) in tissue lysates from the injection sites were analyzed by Western blot (Fig. 5a, n = 5/group/time point). The mean o.d. of VEGF in the Sham groups were 0.09 ± 0.03 as a baseline (combined groups at all time points). The levels in the ICH groups were 0.28 ± 0.02, 0.48 ± 0.24, 0.52 ± 0.24 and 0.68 ± 0.01 at 1d, 3d, 7d and 15d, respectively. In the drug-treated animals, the levels were 0.36 ± 0.19, 0.50 ± 0.12, 0.67 ± 0.14 and 0.82 ± 0.13 in the ICH + NBP10, and were 0.44 ± 0.08, 0.63 ± 0.01, 0.88 ± 0.38 and 0.96 ± 0.15 in the ICH + NBP25 groups, at the above time points, respectively. Thus, there was a trend of increase in the levels of VEGF in the ICH groups relative to Sham control and in the drug-treated relative to vehicle-treated groups, as indicated by a time (P < 0.0001, Df = 3, F = 19) and treatment (P < 0.0001, Df = 3, F = 64) dependent effects. Results from post hoc tests indicated significant differences for all other groups relative to the Sham groups (P < 0.05 to P < 0.0001) except for the Sham vs. ICH groups at 1d. The levels of VEGF between ICH + NBP25 groups and the ICH groups were significantly different (P < 0.05 and P < 0.01) at 7d and 15d. However, no significant differences were reached for the remaining pairs of means among the ICH and ICH + NBP10 and ICH + NBP25 groups (Fig. 5b).

The normalized o.d. of immunoblotted Ang-2 protein in brain lysates were 0.20 ± 0.01 as a baseline for the Sham groups. The levels (mean ± S.D.) were 0.21 ± 0.01, 0.43 ± 0.02, 0.48 ± 0.02, 0.48 ± 0.01 in the ICH groups at 1d, 3d, 7d and 15d, respectively. In the ICH + NBP10 groups, the means were 0.33 ± 0.02, 0.50 ± 0.01, 0.66 ± 0.02 and 0.86 ± 0.01 at the above time points, respectively. The values were 0.36 ± 0.02, 0.60 ± 0.02, 0.91 ± 0.03 and 0.99 ± 0.03 in the ICH + NBP25 groups. There existed a trend of increase in the levels related to the effect of time (P < 0.0001, Df = 3, F = 1715) as well as treatment (P < 0.0001, Df = 3, F = 3441) according to two-way ANOVA analysis. *Post*-*hoc* tests indicated that there existed significant differences for all blood-injected groups in comparison with the Sham groups at all time points (P < 0.001) except for the Sham vs ICH groups at 1d. There were differences for the ICH + NBP10 and ICH + NBP25 groups relative to the ICH groups at all surviving time points (P < 0.001). Furthermore, the levels showed significant differences between the ICH + NBP10 and ICH + NBP25 groups at 1d (P < 0.01), and at 3d, 7d and 15d (P < 0.001) as well (Fig. 5b).

## Discussion

The pathogenesis and pathophysiology underlying ICH are complex. The space-occupying effect of the hematoma along with blood infiltration into the brain parenchyma could trigger multiple cellular and molecular responses around and beyond the lesion site, such as ischemia, blood–brain barrier disruption, oxidative stress and neuroinflammation, which may result in acute and chronic neuronal damage and neurological dysfunction [8, 12, 15]. Clinical trials suggest that vasospasm of larger brain vessels is not the sole contributor to neurological outcome, and intense efforts have been turned to mechanisms of early brain injury that may play a larger role in functional outcome, including neuroinflammation and microvascular dysfunction [47]. Specifically, the structural and functional disruption of neurovascular unit (NVU) may be critically involved in the pathogenesis of cerebral stroke [17]. NVU is structurally related to neuronal and neuroglial cells, as well as microvascular endothelial cells, smooth muscle cells, pericytes and the extracellular matrix [18–22]. Thus, through tightly regulated multidirectional molecular signaling, the NVU is considered to be involved in many key processes critical for functional neurovascular coupling, including via a response of angiogenesis. Thus, the formation of new blood vessels from existing vasculature in the peri-infarct or perihemorrhagic zone is considered important for neurological recovery after ischemic or hemorrhagic cerebral stroke [23–26]. Accordingly, promoting angiogenesis represents a novel therapeutic strategy in clinical management of stroke patients.

Since the approval by the China Food and Drug Administration for the treatment of ischemic stroke, NBP has been clinically used in the management of patients with ischemic cerebrovascular diseases [32–34]. Experimental studies have identified multiple potential neuroprotective effects of this compound, such as increasing blood perfusion to the ischemic area, preventing blood brain barrier disruption, protecting mitochondria, inhibiting nerve cell apoptosis, scavenging oxygen free radicals, suppressing inflammation and reducing cerebral edema in animal models of cerebral ischemia [36–45, 47–49]. It should be noted that precautions were (and still remain) recommended for the prescription of this drug to patients with hemorrhagic stroke because of a concern of re-bleeding side-effect. However, to the best of our knowledge, no data from human clinical or animal studies are yet available to confirm this adverse effect. In this regard, the pharmacological efficacy or toxicity, if any, of NBP on hemorrhagic cerebral stroke, and the mechanism thereof, deserves experimental investigation.

Several rodent models of ICH are commonly used for basic and translational studies on hemorrhagic cerebral stroke in humans, including perforation of cerebral arteries, and intracranial or intracerebral injection of self-blood [50–53]. The effects of the lesions and experimental interventions could be addressed using various batteries of neurobehavioral tests along with different morphological and biochemical measurements. The intra-striatal blood injection model was used in this study to induce ICH, which mimics the internal capsule stroke commonly seen in humans in the clinic. One advantage of this model is that the post-operational animal surviving rate is relatively high comparing to other models with more severe or extensive intracranial bleeding. Unilateral injection of autologous blood into the globus pallidus causes acute neurological deficits largely involving motor dysfunctions contralateral to the injection side in the experimental rats, which could be assessed using the Longa scoring method. Notably, as with other rodent models of cerebral stroke or trauma, neurological deficits in rats subjected to this lesion progress with an initial severe phase followed by a “natural” or spontaneous trend of functional recovery [54]. In the present study, motor deficits in rats subjected to intra-striatal blood injection as indicated by Longa scoring occurred apparently at the 3rd day post operation. The deficits in both the ICH + NBP10 and ICH + NBP25 groups appeared to be milder than that in the vehicle controls as observed from 1 to 15 days post operation, with signs of recovery also occurred earlier in the drug-treated animals (starting as early as the first day post operation). These findings suggest that NBP treatment with a dosage 10-25 mg/kg Bid can promote an earlier functional recovery in rats subjected to the current experimentally induced ICH. It should be noted that the volumes of hematoma remained comparable between the ICH, ICH + NBP10 and ICH + NBP25 groups. Therefore, this drug does not appear to promote the clearance of the injected blood in the brain parenchyma.

As denoted precedingly, neovascularization is stimulated in the brain following ischemic or hemorrhagic insults especially in the periinfarct or perihemorrhagic zone, which may represent a positive neurorepair process for functional neurological recovery [23–26]. Upregulation of many molecules, including some soluble factors, is involved in such a process. The highly glycosylated type I transmembrane glycoprotein CD34 is a molecular marker for neovascularization because it is selectively expressed on the hematopoietic stem or progenitor cells as well as the progenitor or differentiating endothelial cells in humans and other mammals, with its expression down-regulated to undetectable levels in mature vascular cells [55–58]. VEGF is a highly conserved dimer glycoprotein serving as a soluble mitogenic factor specifically for vascular endothelial cells. It helps create new blood vessels during embryonic development, and also is key to the formation of collateral circulation to bypass blocked vessels in diseased conditions in adult organisms. After binding to its receptors VEGFR-1 and VEGFR-2, the tyrosine kinase is activated, resulting in calcium inflow in the cells. This signaling can facilitate the proliferation and differentiation of endothelial cells, and also enhance the permeability of vascular endothelial cells to form temporary extracellular matrix, thereby collectively promoting angiogenesis [59, 60]. In healthy adult brain, VEGF is expressed at low levels and is only sporadically detectable in most brain regions [61]. Following ischemic or hypoxic insults, VEGF expression in the brain or the periphery is upregulated to stimulate endothelial cell proliferation and formation of new blood vessels [62–64]. Ang-2 is another important soluble factor for angiogenesis. This protein is also highly expressed in tissues during embryonic development, and remains expressed at minimal levels in endothelial cells at adulthood under normal conditions [65–67]. Ang-2 can facilitate the response of vascular endothelial cells to VEGF [67]. The synergistic effect of these two factors provides a strong support to neovascularization following various central and peripheral injuries or vascular blockage [61–65]. In addition, both factors can play a pathogenic role in the malignant growth of tumors that depends on blood supply via angiogenesis [66–69].

In the present study, the numerical density of putative new microvascular profiles was increased in the ICH relative to Sham groups post operation during the observation time period. NBP treatments at the two selected doses could increase the amount of these microvascular profiles in the perihemorrhagic zone. The fractional areas of vascular profiles were increased in the ICH relative to Shame groups. While a trend of increase in the vascular area also occurred in the drug-treated groups, the effect between the drug and vehicle-treated groups did not reach statistical significance at most of the time points examined (except for the ICH + NBP25 vs. ICH at 7 and 15d). The lack of a full parallelism between the increases of vascular number and area might be related to the transient expression of CD43 in endothelial cells during angiogenesis, which could limit the increase of the sum area of the new microvasculature. The elevation of VEGF and Ang-2 levels in the ICH relative to Sham groups is consistent with a lesion response [61–67]. Importantly, the levels of these two angiogenic factors were significantly higher in the drug-treated than the vehicle-treated groups at most surviving time points (i.e., VEGF in the ICH vs. ICH + NBP25 groups at 7d and 15d, and Ang-2 in the ICH vs. the ICH + NBP10 and ICH vs. ICH + NBP25 groups at all surviving time points). Together, the morphological and biochemical data from this study support the notion that NBP treatment at 10–25 mg/kg Bid can promote neovascularization in the perihemorrhagic zone in the current model of experimental ICH.

## Conclusions

The present study shows that in a rat model of ICH via intra-striatal injection of autologous blood, NBP administration at 10 and 25 mg/kg Bid can significantly mitigate motor deficits and allow an earlier neurological recovery. Importantly, NBP treatment can promote neovascularization around the lesion site potentially related to the upregulation of VEGF and Ang-2. Clinical use of this drug among patients with cerebral hemorrhage might be beneficial for poststroke neurological recovery.

## Methods

### Animals

Male Sprague–Dawley rats weighting 210 ± 20 g and aged 8–12 weeks-old were obtained from the Dongtang Animal Center of Hunan University of Traditional Chinese Medicine. Rats were maintained in their vivarium under programmed control of temperature (23 ± 2 ^◦^C), humidity (50 ± 5%) and illumination (12/12 h light/dark cycle), with standard rodent chews and water freely accessible during the entire experimental period.

### Surgery and drug administration

ICH was surgically induced according to the method described by Rosenberg et al. [70]. Briefly, rats were anesthetized with sodium pentobarbital at a dosage of 50 mg/kg via intraperitoneal (i.p.) injection, and placed on a stereotaxic apparatus, with craniotomy performed under sterile conditions. Approximately 0.2 ml autologous whole blood was drawn from the femoral artery. Subsequently, 100 μl semi-coagulated blood was injected into the right globus pallidus slowly using the following coordinates: 1 mm posterior to Bregma, 3 mm lateral to the sagittal suture and 5.5 mm below skull surface. The needle was maintained in place for an additional 5 min following the infusion to prevent backflow, and then withdrawn slowly. The skull hole was sealed with bone wax, and the skin incision was closed with sutures. Rats in the Sham group were injected with sterile physiological saline instead of blood following the same surgical procedure. During surgery the animal was placed on a warm water bag to maintain body temperature. After operation, rats were kept warm in a cage under a 60 W incandescent lamp until they fully recovered from anesthesia, followed by an initial verification of the occurrence of neurological deficits using the Longa’s scoring method [54]. The animals that did not exhibit neurological deficits (n = 24) or died (n = 17 in total) during the experiments were discarded from this study.

NBP was purchased from Shijiazhuang Pharmaceutical Co., Ltd (Hebei, China). It was dissolved in soybean oil for oral gavage. The animals were randomly divided into: (1) sham-operated group (Sham group, n = 40) (2) ICH model group receiving soybean oil only as vehicle control (ICH group, n = 40), (3) low dosage NBP treatment animals (ICH + NBP10 group, n = 40) that were subjected to ICH induction and fed with NBP10 at 10 mg/kg/Bid, and (4) moderate NBP dosing group (ICH + NBP25 group, n = 40) consisted of operated animals receiving NBP at 25 mg/kg/Bid. These two dosages were chosen in reference to previous rodent studies [36–40]. NBP was dosed first approximately 4 h before the animals were anesthetized for surgery, and until the day they were euthanatized with a killing dose (100 mg/kg, i.p.) of sodium pentobarbital for terminal brain examination. Behavioral test and brain examination were carried out at 1, 3, 7 and 15 days (d) after operation, respectively.

### Assessment of neurological deficits

Neurobehavioral assessment was conducted on control and drug-treated animals (n = 10/group/time point) according to the Longa’s scoring method [54]. Thus, a score 0 was defined if the animal showed no any movement abnormality. Score 1 was given when the animal could not fully extend its left forelimb. Score 2 was recorded if the animal rotated to the left side while walking. Score 3 was given if the animal dumped to the left side while walking. Score 4 was noted if the animal could not walk on its own, but remained consciously alert.

### Measurement of hematoma volume

Hematoma volume in the brain was estimated using equally spaced coronal cerebral slices (n = 5/group/time point). Thus, after removal from the skull, the brain was serially sliced at the coronal plane with 1 mm intervals. Images of consecutive slices (up to 6) together with a ruler were taken with a digital camera and saved as a TIF file. The area of hematoma in each slice (expressed as A1, A2,…A6) was measured on computer screen at high resolution using the NIH image J software. The total volume of hematoma in each brain was calculated according to the following formula: V = t × (A1 + A2,… + A6) mm^3^, wherein V refers to the volume of the hematoma, t is the thickness of the brain slice, and A1, A2,…A6 are the areas of hematoma measured in consecutive slices.

### Microscopical study of microvasculature

Histology and immunohistochemical labeling with a CD34 antibody, a marker for newly formed blood vessels [55–58], were applied to determine the potential effect of NBP treatment in promoting neovascularization. After behavioral test and brain collection (n = 5/group/time point), a tissue block covering the hematoma and its surrounding region was dissected out from the slice with the most visible bleeding site. Tissue blocks were then fixed in formalin, dehydrated in ascending concentrations of ethanol, permeabilized in xylene and embedded in wax. Paraffin Sections (5 µm-thick) were then prepared, and subjected to immunohistochemistry to visualize CD34 labeled blood vessels.

Immunolabeling was initiated with a process of dewaxing with xylene and rehydration with descending concentrations of ethanol until rinses with phosphate-buffered saline (PBS, 0.1 M, pH = 7.3). Sections were then treated with 5% H_2_O_2_ in PBS for 30 min, and incubated in a PBS buffer containing 5% normal rabbit serum and 0.3% Triton X-100 for 1 h (h). Sections were further incubated with the above buffer solution containing the rabbit anti-mouse CD34 antibody (1:500, ab185732, Abcam Trading Shanghai Company Ltd, Shanghai, China) at 4 °C overnight. On the next day, the sections were reacted with biotinylated rabbit anti-mouse IgG at 1:400 for 1 h, and further with the avidin–biotin complex (ABC) reagents (1:400) (Vector Laboratories, Burlingame, CA, USA) for 1 h. The final immunoreactive product was visualized in PBS with 0.003% H_2_O_2_ and 0.05% 3,3′-diaminobenzidine. Three rinses with PBS were used between the incubations. Sections were then counterstained lightly with toluidine blue, dehydrated with ethanol, cleared with xylene, and coverslippered before microscopical examination.

### Western blot for angiogenic markers

Immunoblotting was used to detect potential changes in the levels of two important angiogenesis related proteins, i.e., VEGF and angiopoietins-2 (Ang-2) [40, 46]. Brains from the rats in the drug-treated and control groups surviving to all timed points were subjected to this analysis (n = 5/group/time point). Following consecutive brain slicing, tissue blocks surrounding the hematoma in the right globus pallidus were excised, carefully isolated, snap-frozen in liquid nitrogen, and stored at − 80 C until use. The samples were then homogenized on ice by sonication in T-PER extraction buffer (Pierce, Rockford, IL, USA) containing a cocktail of protease inhibitors (Roche, Indianapolis, IN, USA). The supernatants were collected following centrifuge of the tissue extracts at 15,000 *g* at 4 °C, with protein concentrations determined using the BCA assay. Supernatants containing equal amount of total protein were run in SDS-polyacrylamide gels. Separated proteins were electrotransferred to Trans-Blot pure nitrocellulose membranes. The membranes were then incubated overnight at 4 °C with a rabbit antibody against recombinant human VEGF (D151121, 1:1000, Sangon Biotech. Co., Ltd. Shanghai, China), a rabbit anti-Ang-2 polyclonal antibody (ab139947, 1:1000, Abcam Trading Shanghai Company Ltd, Shanghai, China), and a rabbit anti-β-actin antibody as loading control (PA1-16889, 1:5000, ThermoFisher Trading Shanghai Company Ltd, Shanghai, China), respectively. The membranes were further reacted with horseradish peroxidase (HRP) conjugated goat anti-rabbit IgG (1:10000; Bio-Rad Laboratories). Immunoblot signal was visualized with the ECL-Plus detection kit according to manufacturer’s instruction (Thermo Fisher Scientific Trading Shanghai Company Ltd, Shanghai, China), followed by X-ray film exposure, film development and image capture using a laser scanner.

### Imaging acquisition and data analysis

CD34 immunolabeled sections were examined on an Olympus (BX60) microscope equipped with a digital camera and an image analysis system (Optronics, Goleta, CA). Putative new blood vessels appearing as funicular or circular hollow profiles were identifiable at low magnification. For morphometry, images (with a total microscopic field area of 0.2 mm^2^) were taken using the 20× objective lens over the areas approximately at the 12, 3, 6, and 9 clock points relative to the center of needle track or the hematoma. Vascular profiles consisted of individual endothelial cells or endothelial cell clusters were counted, whereas larger profiles with a lumen greater than eight red blood cell’s diameter were not included in the counting. The newly formed vascular profiles as defined above were also marked and subjected to fractional area measurements, which were carried out using the NIH image J software. The fractional area of the blood vessels was calculated for each micrograph using square millimeter as the unit area. Data were recorded into Excel Spreadsheets, with averaged values for individual brains, and further, means and standard deviations for individual animal groups, calculated. Images of immunoblotted protein bands were also analyzed using the image J software. The optic densities (o.d.) of the VEGF and Ang-2 bands were standardized to that of the β-actin as internal control, and expressed as density ratios.

### Statistical tests and figure preparation

Experimental data were presented as mean ± standard deviation (mean ± SD). Statistical tests were performed using the GraphPad Prism 5.1 (San Diego, CA, USA) software package. Data were analyzed with two-way ANOVA along with Bonferroni multi-group comparison as post hoc tests (reflecting the effects of time and treatment variables and their reaction), with the minimal level of significant difference set at P < 0.05. All figures were assembled using Photoshop 7.1.

## Data Availability

The datasets used and/or analyzed during the current study are available from the corresponding author on reasonable request.

## Abbreviations

NBP: Dl-3-n-Butylphthalide
ICH: Intracerebral hemorrhage
PHZ: Perihemorrhagic zone
NVU: Neurovascular unit
VEGF: Vascular endothelial growth factor
Ang-2: Angiopoietins-2
NIH: National Institutes of Health
PBS: Phosphate-buffered saline
ABC: Avidin-biotin complex
BCA: Bicinchoninic acid
SDS: Sodium dodecyl sulfate
HRP: Horseradish peroxidase
